# circSORBS1 inhibits lung cancer progression by sponging miR-6779-5p and directly binding RUFY3 mRNA

**DOI:** 10.1186/s12967-024-05423-0

**Published:** 2024-06-24

**Authors:** Haotian Xu, Yue Zheng, Jiaxi Wu, Ruirui Zhang, Qingyun Zhao, Sixian Chen, Wenyi Peng, Dunyu Cai, Yihong Gao, Xingcai Chen, Deqing Li, Shengyi yuan, Gang Li, Aruo Nan

**Affiliations:** 1 School of Public Health, Guangxi Medical University, Nanning, 530021 China; 2 Guangxi Key Laboratory of Environment and Health Research, Guangxi Medical University, Nanning, 530021 China

**Keywords:** Lung cancer, circSORBS1, miR-6779-5p, RUFY3

## Abstract

Lung cancer is the primary cause of cancer-related death worldwide, and its global incidence and mortality rates remain high. The differential expression of circular RNAs (circRNAs) can affect the development of cancer, but the mechanisms by which circRNAs regulate lung cancer progression remain unclear. In this study, we identified circSORBS1, a circRNA that has not been previously described in lung cancer and is significantly underexpressed in lung cancer tissues, blood and cell lines, and the low expression of circSORBS1 correlated with tumour grade and prognosis. In vitro and in vivo functional experiments revealed that circSORBS1 overexpression inhibited cell proliferation and migration while enhancing apoptosis. Mechanistically, circSORBS1 acts as a sponge for miR-6779-5p, indirectly inhibiting RUFY3 mRNA degradation. Simultaneously, it binds to RUFY3 mRNA to enhance its stability. This dual regulatory mechanism leads to an increase in RUFY3 protein levels, which ultimately activates the YWHAE/BAD/BCL2 apoptotic signalling pathway and suppresses lung cancer progression. Our findings not only increase the knowledge about the regulatory pattern of circRNA expression but also provide new insights into the mechanisms by which circRNAs regulate lung cancer development.

**Supplementary Information:**

The online version contains supplementary material available at 10.1186/s12967-024-05423-0.

## Introduction

According to global cancer statistics, lung cancer is one of the most common cancers in the world and the leading cause of cancer deaths [1], with high incidence and mortality rates, especially in developing countries [2]. Lung cancer is mainly categorized into small cell lung cancer and non-small cell lung cancer [3], and its risk factors include smoking, environmental exposure (e.g., second-hand smoke, asbestos, and air pollution), and genetic factors. Surgery, radiotherapy, chemotherapy and targeted therapy are the main treatments [4]. However, most patients are in advanced stages at the time of diagnosis and miss the best opportunity for surgical treatment. In recent years, the development of targeted therapy and immunotherapy has brought new hope to lung cancer patients [5], but the lack of targets and high mutation rate are still challenges for treatment [6–8]. Therefore, it is particularly important to explore the molecular mechanism of lung cancer and identify potential molecular targets for treatment.

circRNAs are a class of functional RNAs that are formed by reverse splicing or jumping of precursor mRNAs into circular structures without 5′ caps or 3′ polyadenylation tails and are widely and stably expressed in a variety of eukaryotic cells [9, 10]. circRNAs are highly conserved and tissue specific and show great potential for cancer diagnosis and therapy. Previous studies have shown that aberrant expression of circRNAs plays a key role in tumours [11–13]. circRNAs act as miRNA sponges, interact with proteins, encode proteins and peptides, and regulate gene expression [10]. For example, circRNA_0025202 acts as a miR-182-5p sponge and promotes FOXO3a expression, thereby inhibiting breast cancer progression [14]. In addition, circLAMA3 targets MYCN mRNA and promotes its degradation, reducing MYCN expression in bladder cancer cells [15]. Although the molecular mechanisms and relationships among circRNAs, miRNAs and mRNAs have been reported [16], further studies are needed to elucidate the circRNA regulatory network.

circRNAs are involved in cellular events such as programmed death, autophagy and glycolysis through multiple pathways to regulate tumour development. Apoptosis is a form of programmed death that plays an important role in tumorigenesis and development [17]. Apoptotic pathways are categorized into extrinsic (death receptor pathway) and intrinsic (mitochondrial pathway) pathways [18]. The extrinsic pathway is mainly triggered by tumour necrosis factor (TNF) family receptors that interact with ligands to activate the caspase-8 protein and trigger apoptosis [17]. The intrinsic pathway involves mitochondrial events that induce the opening of the mitochondrial permeability transition pore (mPTP) through positive or negative signalling, releasing proapoptotic proteins and cytochrome C. BCL2 proteins are the key regulators of the intrinsic pathway and include multiple members [18], such as BCL2 and BCL-XL, which are highly expressed in tumour cells and defend against apoptosis by regulating mitochondrial membrane potential and cytochrome C release, thus altering cell fate [19]. Studies targeting the high expression of BCL2 in tumour cells have revealed that these interactions may further regulate the expression of BCL2 proteins and affect apoptosis [20, 21]. Therefore, an in-depth study of the mechanism of interaction of the BCL2 protein with other factors may help elucidate the molecular mechanism of apoptosis regulation and provide a theoretical basis for the development of more effective tumour therapeutic regimens.

In this study, we constructed circRNA differential expression profiles by high-throughput sequencing and found that circSORBS1 was significantly downregulated in lung cancer. In vitro and in vivo functional studies revealed that circSORBS1 can inhibit the development of lung cancer through multiple mechanisms, including cell viability, proliferation, apoptosis, and migration. Subsequently, we found that circSORBS1 acts as a miR-6779-5p sponge and indirectly inhibits RUFY3 mRNA degradation, while also directly binding to RUFY3 mRNA and enhancing mRNA stability, which in turn increases RUFY3 protein expression, activates the YWHAE/BAD/BCL2 apoptosis signalling pathway, and inhibits lung cancer progression. In summary, we elucidated that circSORBS1 can regulate apoptosis and change cell fate via epigenetic regulation, which not only provides new insights into the mechanism of lung cancer development but also provides new potential molecular targets for lung cancer screening and diagnosis.

## Materials and methods

### Cell culture

Normal human bronchial epithelial cells (BEAS-2B) were purchased from the American Type Culture Collection (ATCC), lung cancer cell lines (H1299, H226, H460, H2170, and A549) were purchased from the Typical Culture Collection Center of the Chinese Academy of Sciences (Shanghai, China), and 293T cells were purchased from Saikou Biotechnology (Guangzhou, China). BEAS-2B cells were cultured in bronchial epithelial cell growth medium (BEGM; Lonza, CC-3171). H1299, H226, H460 and H2170 cells were cultured in RPMI-1640 medium supplemented with 10% foetal bovine serum (Gibco, 10099141C) and 1% penicillin‒streptomycin. A549 cells were cultured in 89% Ham's F12 medium supplemented with 10% foetal bovine serum (Gibco, 10099141C) and 1% penicillin‒streptomycin. The 293T cells were cultured in DMEM supplemented with 10% foetal bovine serum (Gibco, 10099141C) and 1% penicillin‒streptomycin. All the cell lines were cultured in a cell culture incubator at 37 °C with 5% CO_2_.

### Tissue samples

The 110 pairs of tissue samples and 23 pairs of plasma samples used in this study were obtained from the First Affiliated Hospital of Guangxi Medical University. All participants providing samples used in the study signed an informed consent form. All experiments were performed in accordance with the Declaration of Helsinki and approved by the Ethics Committee of the First Affiliated Hospital of Guangxi Medical University.

### Experimental animals

Four-week-old female specific pathogen-free (SPF)-grade nude mice were acquired from Guangxi Medical University Laboratory Animal Center. Animal research was conducted in accordance with international guidelines, and the management of the experimental animals was in accordance with the basic operational requirements for experimental animals. All animal experiments were approved by the Experimental Animal Ethics Committee of Guangxi Medical University.

### RNA extraction and RT‒qPCR

Total RNA was isolated using TRIzol reagent (Invitrogen, 15596018) and quantified using a Nanodrop One spectrophotometer (Thermo Fisher Scientific, ND-ONEC-W) according to the product instructions. A GoScript™ Reverse Transcription System Kit (Promega, A5002) was used for reverse transcription of circRNAs and mRNAs. A Mir-X™ First Strand Synthesis Kit (Takara, 638315) was used for reverse transcription of the miRNAs. qPCR was performed on an Applied Biosystems QuantStudio 7 Flex Real-Time PCR System (Thermo Fisher Scientific) using GoTaq qPCR Master Mix (Promega, A6001). U6 served as an internal reference for miRNAs, and β-actin and GAPDH served as internal references for circRNAs and mRNAs, respectively. The primers used for RNA analysis are shown in Table S1.

### Cell transfection

Overexpression plasmid transfection was performed using Lipofectamine^®^ 3000 (Invitrogen, L3000015). The cells were collected, and 3 × 10^5^ cells were seeded into 6-well plates and cultured to 80% confluence. The plasmid concentration in each well was changed to 2.5 µg after 12 h, after which the cells were incubated for 48 h for subsequent experiments. Small interfering RNA (siRNA) transfection was performed using the riboFECT CP Transfection Kit (RiboBio, C10511-05). All targeting and control siRNA sequences were synthesized by Guangzhou RiboBio Biotechnology Co. A total of 3 × 10^5^ cells were seeded into 6-well plates, and when the cell confluence reached 30%, siRNA (20 nM) was transfected into the cells in each well prior to incubation for 48 h for subsequent experiments.

### Establishment of stably transfected circSORBS1-expressing cell lines

The circSORBS1 stable overexpression plasmid was constructed using a lentiviral vector (V54B pLV-circRNA-has). Lentiviral packaging was performed in 293T cells. The viral supernatant was obtained, centrifuged and filtered, and added dropwise to H226 and H1299 cells for lentiviral infection; the cells were then incubated for more than 48 h. Fluorescence signals were evaluated via fluorescence microscopy (AMG EVOS, Mill Creek, WA, USA). Single-cell colonies were screened for stably transduced cells. (The abbreviation “OE” represents transduced cell lines with stable overexpression, and the abbreviation “oe” represents transfected cell lines with transient overexpression.)

### Plasmid extraction

The RUFY3 and BCL2 plasmids and the corresponding empty vectors (pcDNA3.1) were purchased from Hunan FengHui Science and Technology Co. The plasmid sequences were verified by sequencing. Transformed DH5α cells were added to Luria–Bertani medium (LB) (5 g/L yeast powder [Solarbio, Y8020], 10 g/L NaCl [Aladdin, C111533], and 5 g/L tryptone [Solarbio, T8490]) and incubated on a shaker for 14 h. Plasmid extraction was performed using the Plasmid Midi Kit (QIAGEN, 12145) according to the manufacturer's instructions.

### Verification of the circular structure of circSORBS1

After total cellular RNA was collected, 3 U/µg RNase R was added. The products were digested in a 37 °C water bath for 10 min, after which RT‒qPCR was performed. Cellular DNA was collected using a gDNA extraction kit (Invitrogen, K1820) and was washed and purified several times for backup samples. Convergent primers and divergent primers for circSORBS1 were designed. After RNase R digestion, the gDNA samples were subjected to PCR amplification and then agarose gel electrophoresis.

### Fluorescence in situ hybridization

Analysis of the subcellular localization of circSORBS1 was performed using an RNA FISH kit (RiboBio, C10910). A circSORBS1-specific FISH probe was designed, and the cells were seeded on slides and allowed to grow to 20% confluence. The cells were then fixed, permeabilized, dehydrated, subjected to probe hybridization overnight at 42 °C, washed with 2 × saline sodium citrate (SSC), and stained with 4′,6-diamidino-2-phenylindole (DAPI). The subcellular localization of circSORBS1 was then analysed from images acquired using an LSM800 confocal microscope (Zeiss).

### Nuclear–cytoplasmic fractionation

Nuclear–cytoplasmic fractionation was performed using the PARIS™ kit (Invitrogen, AM1921). First, 3 × 10^6^ cells were collected, and the nuclear and cytoplasmic fractions were separated using a kit. β-Actin was used as the cytoplasmic marker, and U6 was used as the nuclear marker. The distribution of circSORBS1 in the nucleus and cytoplasm was evaluated using RT‒qPCR.

### mRNA stability assay

Specific numbers of cells were seeded in 6-well plates for culture. When the cells reached ~ 90% confluence, actinomycin D (Act D: 2 μg/mL) was added to the cells. Cellular RNA was extracted after 0, 4, 8, 12, and 24 h of treatment, and circSORBS1 and SORBS1 expression was measured via RT‒qPCR.

### Cell viability assay

The cell viability assay was performed using a Cell Counting Kit-8 (CCK-8, Dojindo, CK04). A total of 1 × 10^3^ cells were seeded in 96-well plates and transfected when the cell confluence reached 50%. Forty-eight hours after transfection, CCK-8 reagent was mixed with culture medium at a ratio of 1:10, and the mixture was added to the cells in the blank, control and experimental groups. The absorbance was measured at 450 nm after incubation for 2 h in a 37 °C incubator.

### 5-Ethynyl-2-deoxyuridine (EdU) incorporation assay

A total of 5 × 10^3^ cells were seeded in 96-well plates and transfected at a suitable confluence. The cell proliferative capacity was assayed using a Cell-Light EdU Apollo 567 In Vitro Kit (RiboBio, C10310-1). EdU incorporation, cell fixation, Apollo staining and Hoechst 33342 staining were performed according to the manufacturer's instructions, and the cells were imaged using an EVOS^®^ FL automated imaging system. Finally, the cell proliferative capacity was analysed using ImageJ.

### Cell cycle and apoptosis analyses

A total of 2 × 10^6^ cells were seeded in 6-well plates and transfected at 30–40% confluence. Cell cycle analysis was performed using a cell cycle detection kit (KeyGen Biotech, KGA512). In brief, the cells were fixed at − 20 °C overnight. After digestion with RNase A for 30 min, the cells were stained with PI and counted with a CytoFLEX flow cytometer (Beckman Coulter). Apoptosis analysis was performed using an Annexin V-FITC/PI Double Staining Cellular Modulation Detection Kit (KeyGen Biotech, KGA107). After double staining with FITC and PI, cell counting was performed using a CytoFLEX flow cytometer (Beckman Coulter), and the ratio of early apoptotic to late apoptotic cells was calculated.

### Wound healing assay

A total of 2 × 10^6^ cells were seeded into 6-well plates and transfected at 30–40% confluence. After 48 h of transfection, straight and uniform scratches (forming a grid of nine squares) were made in the cell layer on the bottom surface of each well using a 200 μL pipette tip. Then, 0 h and 24 h after the scratches were made, images were acquired using a microscope (Olympus, Japan), the cells were counted, and the data were analysed.

### Transwell migration assay

A total of 2 × 10^6^ cells were seeded in 6-well plates and transfected at 30–40% confluence. Serum-free medium was added to the upper migration chambers (Corning, USA) and equilibrated for 2 h. Then, 700 μL of complete medium was added to the lower chambers, and 400 μL (containing 1 × 10^4^ cells) of a cell suspension (in basal medium) was added to the upper chambers. After 48 h of incubation, the cells were fixed with methanol and stained with crystal violet. Unmigrated cells within the chambers were removed by wiping, and the remaining cells were imaged using the EVOS^®^ FL Automated Imaging System. ImageJ was used to determine the number of migrated cells.

### Subcutaneous tumour formation assay in nude mice

Ten 4-week-old female BALB/c nude mice were randomly divided into two groups: a group with stable overexpression of circSORBS1 and a control group. After one week of acclimatization in a specific pathogen-free (SPF)-grade environment, 3 × 10^6^ cells were injected into the right dorsal surface of the nude mice. The tumour size was measured every 4 days using Vernier callipers. The volume was calculated using the formula length × height^2^ × 0.5. After 16 days, the nude mice were euthanized, and the tumours were collected. Some of the tumours were fixed with 4% PFA, while others were stored at − 80 °C and frozen for subsequent immunohistochemical (IHC) and Western blot analyses. All animal experiments in this study were approved by the Ethics Committee of Guangxi Medical University.

### Western blot analysis

Total cellular protein was obtained by lysing cells using an in-house-prepared cell lysis buffer (containing 10 mM Tris–HCl [pH 7.4], 1% SDS, and 1 mM Na_3_VO_4_). Protein concentrations were determined using the Pierce™ BCA Protein Assay Kit (Thermo Fisher Scientific, 23227). Proteins in the samples were separated by 8–10% sodium dodecyl sulfate‒polyacrylamide gel electrophoresis (SDS‒PAGE) and transferred to polyvinylidene difluoride (PVDF) membranes over a 4.5 h period at a constant voltage of 25 V. The membranes were blocked for 1 h using 5% skim milk and incubated with primary antibodies overnight at 4 °C. Subsequently, chemiluminescence imaging was performed using a Clinx S6 system, and the grayscale values of the protein bands were analysed using ImageJ. The antibodies used in this study were as follows: anti-RUFY3 (Affinity, #DF15498), anti-BCL2 (Affinity, AF6139), anti-BAD (ProteinTech, 67830-1-IG), anti-YWHAE (ProteinTech, 11648-2-AP), anti-rabbit IgG (Cell Signaling Technology, 8890S), and anti-mouse IgG (Cell Signaling Technology, 4408S).

### RNA antisense purification assay

An RAP kit (BersinBio, Guangzhou, China, Bes5103) was used in this study. Specific procedures were performed according to the manufacturer’s instructions. Briefly, target RNAs were enriched using circSORBS1-specific biotin-labelled probes, and other RNAs and proteins that interact with these target RNAs were adsorbed onto magnetic beads and then purified by elution. The products were subsequently analysed using RT‒qPCR.

### Dual-luciferase reporter assay

The circSORBS1 and RUFY3 wild-type (WT) and mutant (MUT) vectors were constructed by Hunan Fenghui Biotechnology Co., Ltd. (China). A total of 4 × 10^5^ cells were seeded in 6-well plates and cultured for 12 h. The cells were cotransfected with the wild-type or mutant plasmids or with the miR-6779-5p mimic or mimic NC, and after a total of 36 h of cotransfection, firefly and Renilla luciferase activities in the cells were measured using the Dual-Luciferase Reporter Gene Assay Kit (Promega, WI, USA, E1901). Statistical analysis was performed according to the kit instructions.

### Co-immunoprecipitation (Co-IP) assay

Co-IP assays were performed using a coimmunoprecipitation (Co-IP) kit (BersinBio, Guangzhou, China, Bes3011) following the manufacturer’s instructions. The cells were first collected and lysed, the selected antibody (5 μg) was mixed with the cell lysates, and the mixtures were incubated first at 4 °C overnight and then with protein A/G magnetic beads for 3 h at room temperature. The enriched proteins on the magnetic beads were finally eluted and denatured at 100 °C for Western blot analysis.

### Immunohistochemical (IHC) analysis

Tissues were fixed, embedded in paraffin and sectioned. Then, the sections were incubated with specific antibodies, including anti-RUFY3 (1:100), anti-BCL2 (1:100), anti-Ki67 (1:1,000), anti-E-cadherin (1:500), anti-CDK4 (1:100), anti-BAX (1:500), anti-BAD (1:500) and anti-YWHAE (1:200) primary antibodies, and the corresponding secondary antibodies. This incubation step was followed by haematoxylin and eosin (H&E) staining (Solarbio, China). Images were acquired under a light microscope at 200 × and 400 × magnification (Leica, Mannheim, Germany).

### Immunofluorescence (IF) analysis

Cells were seeded into 12-well plates containing coverslips and cultured to 80% confluence. The cells were then fixed and incubated overnight at 4 °C with the appropriate primary antibody. Afterwards, the cells were incubated for 2 h with the appropriate secondary antibody in the dark. Finally, nuclear staining was performed by adding DAPI (Boster Biological Technology, AR1176). Fluorescence imaging was performed using an LSM800 confocal microscope (Zeiss, USA). The primary and secondary antibodies used in this study were as follows: anti-RUFY3 (Affinity, DF15498), anti-BCL2 (Affinity, AF6139), anti-BAD (Boster, BA0315-2), anti-YWHAE (ProteinTech, Inc. 11648-2-AP), anti-rabbit IgG (Cell Signaling Technology, 8890S), and anti-mouse IgG (Cell Signaling Technology, 4408S).

### Statistical analysis

All experiments were repeated three times, and the experimental data are expressed as the mean (x̄) ± standard deviation (SD). For comparisons between two sets of measurement data, the t test (for normally distributed data) or the Wilcoxon rank-sum test (for nonnormally distributed data) was used. To assess correlations between variables, Pearson correlation analysis (for normally distributed data) or Spearman correlation analysis (for nonnormally distributed data) was used. Statistical analyses were performed using SPSS 25.0 (IBM, Chicago, USA), and graphs were created using GraphPad Prism 9.0 (San Diego, USA). All tests were two-tailed, with *P* < 0.05 indicating a statistically significant difference. ns, not significant; *P < 0.05; **P < 0.01.

## Results

### The circRNA circSORBS1 is significantly downregulated in lung cancer

**Fig. 1:**
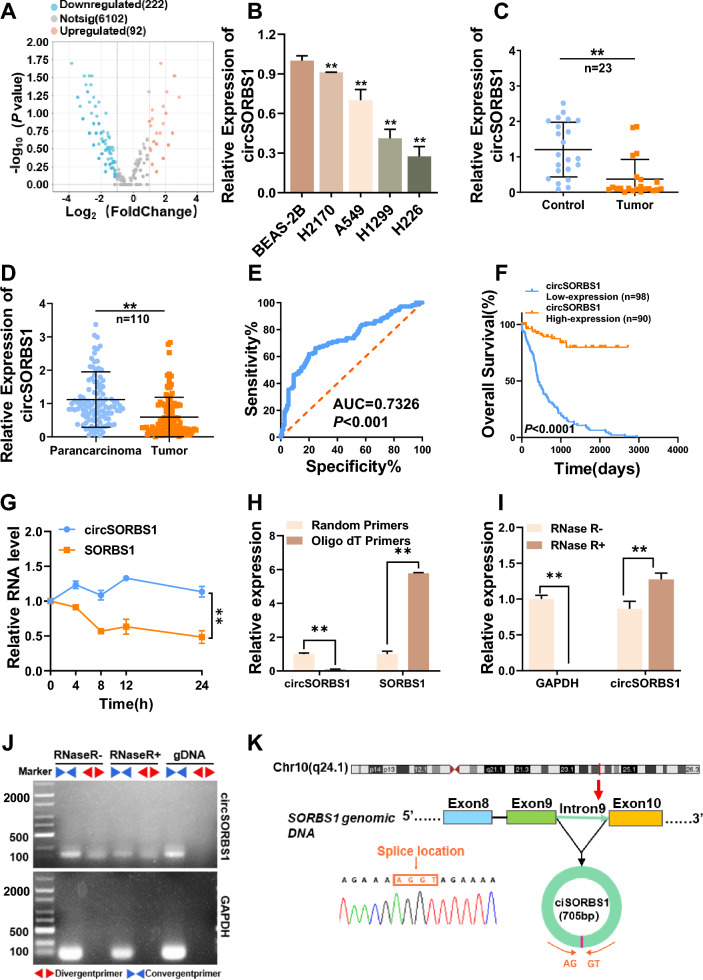
The circRNA circSORBS1 is significantly downregulated in lung cancer. **A** circRNA high-throughput sequencing volcano map, in which 222 genes were downregulated and 92 genes were upregulated. **B** qPCR detection of circSORBS1 expression in human bronchial mucosal epithelial cells (BEAS-2B) and lung cancer cell lines (H2170, H460, A549, H226, H1299). circSORBS1 expression was downregulated in lung cancer cell lines compared to that in BAES-2B cells. **C** qPCR detection of circSORBS1 expression in the serum of 23 healthy individuals (control) versus 23 lung cancer patients (tumour). **D** qPCR detection of circSORBS1 expression in 110 pairs of lung cancer (tumour) and paracancerous (paradancerous) tissues. **E** circSORBS1 ROC curves in population-based lung cancer and paraneoplastic tissues. **F** Survival curves of circSORBS1 after matching with the UCSC database data. **G** Act D stability assay in H1299 cells. **H** Total RNA from H1299 cells was reverse transcribed with random primers and oligo(dT) Primers, respectively. qPCR was performed to compare the reverse transcription efficiency of SORBS1 and circSORBS1. **I** qPCR detection of circSORBS1 expression in samples before and after RNase R treatment, with GAPDH used as a linear control. **J** H1299 cells were divided into two groups: one group was not treated with RNase R, and one group was treated with RNase R. RNA was extracted from the two groups, and the total gDNA of H1299 cells was extracted at the same time. Then, the three groups of samples were subjected to circSORBS1 dispersion and polymerization primer amplification, and the amplified primers were subjected to agarose gel electrophoresis. **K** Schematic diagram of the circSORBS1 gene location and Sanger sequencing results

To explore the biological functions and molecular mechanisms of circRNAs in lung cancer and to identify potential molecular targets for treatment, we performed RNA high-throughput sequencing of lung cancer and paracancerous tissue samples to construct circRNA differential expression profiles (Fig. 1A). Kyoto Encyclopedia of Genes and Genomes (KEGG) pathway enrichment analysis of the 314 differentially expressed circRNAs revealed that the differentially expressed circRNAs were significantly associated with multiple malignancy-related pathways (Figure S1A). The most significantly downregulated circRNA among the 222 downregulated circRNAs, circSORBS1, was subsequently identified. RT‒qPCR analysis showed that circSORBS1 expression was downregulated in lung cancer cell lines (H2170, A549, H1299, and H226) compared to BEAS-2B cells (Fig. 1B). Further measurement of circSORBS1 expression in lung cancer tissue samples and plasma samples from patients with lung cancer revealed that circSORBS1 expression was also significantly downregulated in both types of samples (Fig. 1C, D). The diagnostic efficacy of circSORBS1 in lung cancer screening was evaluated by receiver operating characteristic (ROC) curve analysis, which revealed an area under the curve (AUC) of 0.7326 and a *P* value of < 0.001, suggesting that circSORBS1 has the potential to be a diagnostic indicator for lung cancer (Fig. 1E). Survival analysis revealed that patients with low circSORBS1 expression had worse overall survival than patients with high circSORBS1 expression, suggesting that circSORBS1 expression is closely related to prognosis in lung cancer patients (Fig. 1F).

We searched the UCSC Genome Browser human gene website and found that circSORBS1 is an intronic circRNA generated from intron 9 of the SORBS1 pre-mRNA via back-splicing and has a length of 705 nucleotides (nt). We designed specific primers based on the nucleotide sequence at the back-splicing site of circSORBS1 and performed qPCR and Sanger sequencing to clarify the circularization site (Fig. 1K). Next, to verify the circular structure of circSORBS1, we first extracted total cellular RNA, reverse transcribed the RNA using oligo(dT) primers and random primers, and performed qPCR. Compared with the reverse transcription products obtained with the random primers, the reverse transcription products obtained with the oligo(dT) primers contained almost no circSORBS1, suggesting that almost no circSORBS1 could be amplified with the random primers (Fig. 1H). Subsequently, the extracted total RNA was treated with an enzyme that can digest linear RNA (RNase R), and then qPCR was performed. The results showed that circSORBS1 was more resistant to digestion by RNase R than was linear mRNA (represented by GAPDH) (Fig. 1I). Next, we designed specific divergent and convergent primers based on the structural features of circSORBS1, performed PCR using three sets of samples—samples without RNase R treatment, samples with RNase R treatment, and gDNA samples—and then performed agarose gel electrophoresis. The product amplified with the divergent primers (i.e., circRNA) was more resistant to RNase R digestion than was the product amplified with the convergent primers (i.e., linear RNA) (Fig. 1J). Finally, to further assess the stability of circSORBS1, an RNA stability assay was performed, and the results showed that circSORBS1 was more stable and had a longer half-life than linear SORBS1 mRNA (Fig. 1G). Based on the above experiments, circSORBS1 was identified to have a closed loop structure.

### circSORBS1 inhibits lung cancer development in vitro

**Fig. 2:**
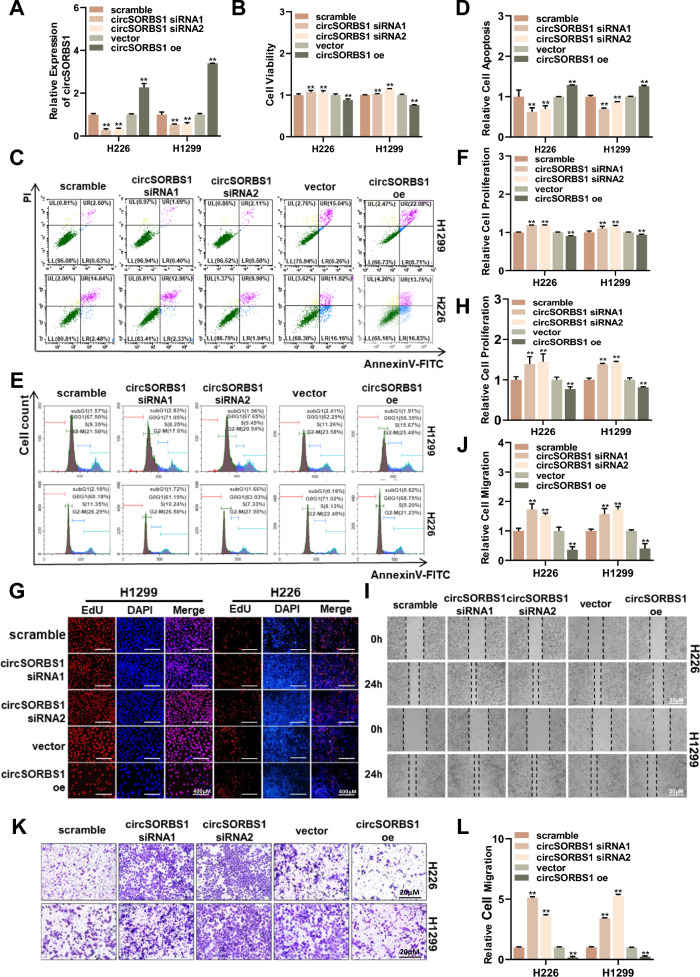
circSORBS1 inhibits lung cancer development in vitro. **A** qPCR detection of the transient silencing and overexpression efficiency of circSORBS1 in H1299 and H226 cells. **B** Cell viability was assessed using the CCK-8 assay in H1299 and H226 cells. **C**, **D** Flow cytometric detection of apoptosis and statistical analysis of H1299 and H226 cells. **E**, **F** Flow cytometry detection of cell proliferation and statistical analysis of H1299 and H226 cells. **G**, **H** Results from the EdU assay for cell proliferation and statistical analysis of H1299 and H226 cells. **I**, **J** Wound healing assay to detect cell migration and its statistical results in H1299 and H226 cells. **K**, **L** Transwell assay to detect cell invasiveness and its statistical results in H1299 and H226 cells

To explore the biological function of circSORBS1 in lung cancer, we performed KEGG pathway enrichment analysis of the mRNAs downstream of circSORBS1 and found that circSORBS1 was significantly associated with multiple cancer-related signalling pathways, suggesting that circSORBS1 has the potential to regulate lung cancer development (Figure S1B). To further explore the biological functions of circSORBS1, we designed and synthesized circSORBS1-specific siRNAs and constructed a circSORBS1 overexpression plasmid to establish a circSORBS1 transient silencing and overexpression experimental system (Fig. 2A; Figure S2A). After successful establishment of the circSORBS1 transient transfection system, we performed functional studies of cell viability, apoptosis, proliferation, migration and other biological processes. We transiently silenced or overexpressed circSORBS1 in H1299 cells and H226 cells, and cell viability was determined using a CCK-8 assay. Cell viability was increased by silencing circSORBS1 and decreased by overexpressing circSORBS1 (Fig. 2B). Subsequently, apoptosis was evaluated by flow cytometry, and the results showed that apoptosis decreased after circSORBS1 silencing and increased after circSORBS1 overexpression (Fig. 2C, D). Cell proliferation was evaluated by flow cytometry and an EdU incorporation assay, which revealed that cell proliferation was enhanced after silencing circSORBS1 and decreased after overexpressing circSORBS1 (Fig. 2E–H). To more comprehensively elucidate the biological function of circSORBS1, we also performed scratch and Transwell assays to evaluate cell migration capacity. Silencing circSORBS1 promoted cell migration, and overexpressing circSORBS1 inhibited cell migration (Fig. 2I–L). In summary, we found that circSORBS1 significantly inhibited the development of lung cancer in vitro, leading us to further test the biological function of circSORBS1 in vivo.

### circSORBS1 inhibits lung cancer development in vivo

**Fig. 3:**
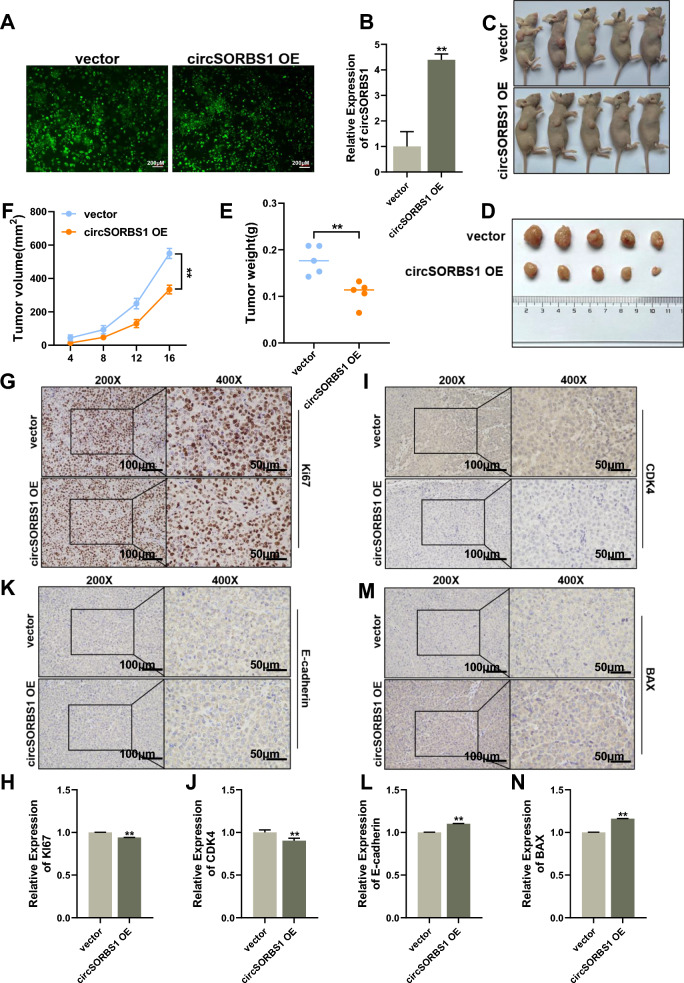
circSORBS1 inhibits lung cancer development in vivo. **A** Construction of a circSORBS1 stable overexpression cell line by lentiviral transfection of H226 cells. **B** qPCR detection of circSORBS1 overexpression efficiency in H226 cells stably overexpressing circSORBS1. **C**, **D** Nude mouse tumour formation experiments and tumour size determination. **E** Statistical analysis of tumour weight in nude mice. **F** Growth of the nude mice. **G**–**N** Immunohistochemical analysis of the protein expression of Ki67, CDK4, BAX, and E-cadherin in nude mouse tumours

In the above experiments, we found that circSORBS1 can inhibit lung cancer development in vitro. Subsequently, we performed subcutaneous tumour formation assays in nude mice to investigate the biological function of circSORBS1 in vivo. First, we constructed a cell line with stable circSORBS1 overexpression via lentiviral transduction (Fig. 3A, B). After successful construction of this cell line, circSORBS1 control and circSORBS1-overexpressing cells were injected subcutaneously into the right dorsal surface of nude mice. The growth status of the nude mice was closely observed, and the body weight and tumour volume were recorded. After the formation of subcutaneous tumours, the nude mice were sacrificed following 16 days of observation, and the tumour masses were removed, weighed and photographed (Fig. 3C, D). Statistical analysis of the tumour volume during tumour growth and the final tumour weight revealed that stable overexpression of circSORBS1 reduced tumour volume and slowed tumour growth relative to the corresponding parameters in the control group (Fig. 3E, F). To further explore the biological functions of circSORBS1 in vivo, we performed immunohistochemical experiments and found that the expression levels of the proliferation-related protein Ki67 and the cell cycle-related protein CDK4 were decreased and that the expression levels of the apoptosis-related protein BAX and the antimigratory/invasive protein E-cadherin were increased in tumour tissues stably overexpressing circSORBS1 (Fig. 3G–N). These results show that circSORBS1 significantly inhibited the development of lung cancer in vivo.

### circSORBS1 acts as a miR-6779-5p sponge and targets RUFY3 mRNA, promoting its stabilization

**Fig. 4:**
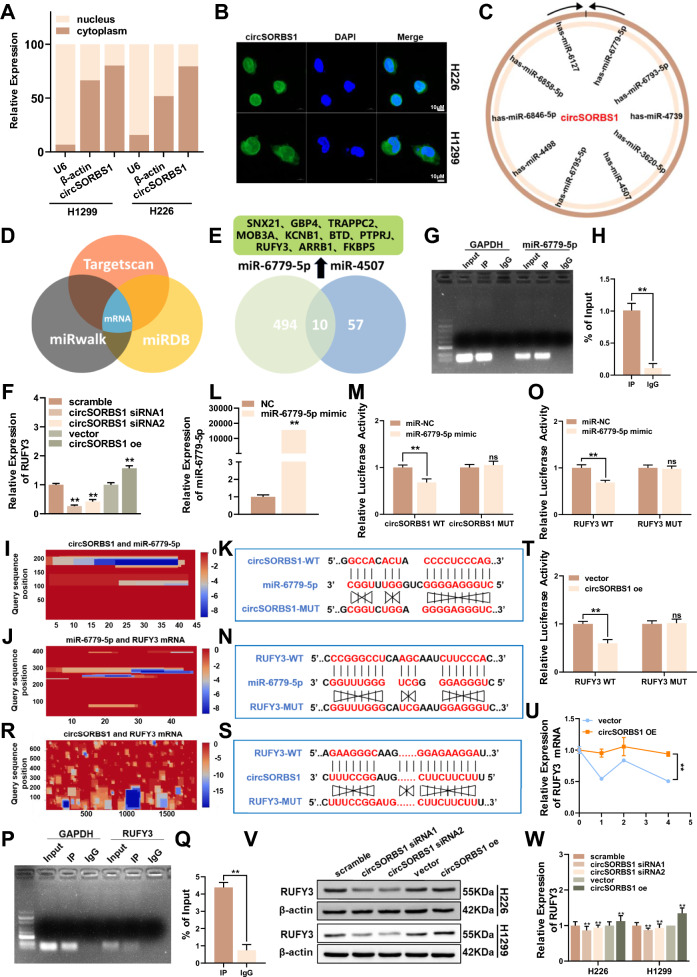
circSORBS1 acts as a miR-6779-5p sponge and targets RUFY3 mRNA, promoting its stabilization. **A** Nucleoplasmic separation experiments were performed in H1299 and H226 cells to detect the expression level of circSORBS1 in the nucleoplasm, with U6 as the nuclear positive control and β-actin as the cytoplasmic positive control. **B** FISH was used to detect the nucleoplasmic distribution of circSORBS1 in H226 and H1299 cells. **C** Prediction analysis of the expression of the miRNAs downstream of circSORBS1 using the circMine website. **D**, **E** Comprehensive prediction by the TargetScan, miRWalk, and miRDB databases was performed to screen 10 downstream mRNAs. **F** RUFY3 mRNA was screened from 10 mRNAs by qPCR. **G**, **H** Design of circSORBS1-specific RAP probes and agarose gel electrophoresis results after RT‒qPCR amplification of the enriched RNA products. **I**, **J** Predicted maps of the binding sites of circSORBS1 to miR-6779-5p and of miR-6779-5p to RUFY3 mRNA via the IntaRNA website. **K** Schematic diagram of the mutation sites in the circSORBS1 and miR-6779-5p dual-luciferase reporter gene plasmid vector. **L** Transfection efficiency of miR-6779-5p mimics detected by qPCR. **M** Analysis of the results of experiments in which circSORBS1 was transfected with the miR-6779-5p dual-luciferase reporter gene. **N** Schematic diagram of the mutation sites in the miR-6779-5p and RUFY3 mRNA dual-luciferase reporter gene plasmid vectors. **O** Analysis of the effects of miR-6779-5p combined with RUFY3 mRNA in dual-luciferase reporter assays. **P**, **Q** Design of circSORBS1 antisense RAP experimental probes, qPCR experiments and agarose gel electrophoresis for the detection of pulled-down RUFY3 mRNA after RT‒qPCR amplification of the pulled-down RNA products. **R** Prediction of the circSORBS1 and RUFY3 mRNA binding sites via the IntaRNA website. **S** Schematic diagram of the mutation site of RUFY3 and circSORBS1 mRNA used for the dual-luciferase reporter gene assay. **T** Analysis of the results of the dual-luciferase reporter gene experiment for circSORBS1 with RUFY3 mRNA. **U** Act D treatment after overexpression of circSORBS1 and qPCR detection of RUFY3 mRNA half-life. **V**, **W** circSORBS1 transient overexpression and silencing were followed by Western blot analysis of RUFY3 protein expression levels and grey value analysis

Through the above functional experiments, we found that circSORBS1 can inhibit lung cancer development both in vivo and in vitro. To clarify the molecular mechanism through which this circRNA inhibits lung cancer development, we performed FISH and nuclear–cytoplasmic fractionation and found that circSORBS1 was distributed mainly in the cytoplasm (Fig. 4A, B). Previous studies report that circRNAs in the cytoplasm play regulatory roles mainly by acting as miRNA sponges and binding to proteins. We performed a prediction analysis using the catRAPID website and found that circSORBS1 has the potential to bind to AGO2 (Figure S2B). Subsequently, we predicted miRNAs downstream of circSORBS1 through the circMine website and identified the two upregulated miRNAs with the strongest binding ability (hsa-miR-6779-5p and hsa-miR-4507) (Fig. 4C). The mRNA targets of these two miRNAs were subsequently comprehensively predicted via three databases (TargetScan, miRWalk, and miRDB), and 10 mRNAs were found to be significantly associated with these two miRNAs (Fig. 4D, E). qPCR revealed that after transient silencing or overexpression of circSORBS1, the changes in the mRNA levels of GBP4, RUFY3, and SNX21 were consistent with the expected changes (Fig. 4F; Figure S2C, D). A review of the related literature and KEGG pathway analysis revealed that RUFY3 is closely related to tumorigenesis and tumour development; therefore, we selected RUFY3 for subsequent experimental investigation. We designed a specific RAP probe based on the circSORBS1 sequence and performed an RAP assay. The results suggested that circSORBS1 binds directly only to miR-6779-5p (Fig. 4G, H). The binding sites between circRNAs, miRNAs, and mRNAs were predicted through the online IntaRNA database, and a heatmap showing the possible binding regions was generated (Fig. 4I, J). To further explore the relationship between circSORBS1 and miR-6779-5p, we constructed circSORBS1 wild-type and mutant dual-luciferase reporter vectors (Fig. 4K). The efficiency of the designed miR-6779-5p mimic was also evaluated (Fig. 4L). After miR-6779-5p overexpression, a dual-luciferase reporter assay was then performed. The luciferase activity in circSORBS1-WT-transfected cells was lower than that in cells transfected with the NC mimic, whereas no significant change in luciferase activity was observed in circSORBS1-MUT-transfected cells, indicating the direct binding of circSORBS1 to miR-6779-5p (Fig. 4M). Moreover, to explore the binding relationship between miR-6779-5p and RUFY3, we constructed RUFY3 wild-type and mutant vectors based on the binding sequence of miR-6779-5p in RUFY3 mRNA (Fig. 4N) and performed a dual-luciferase reporter assay. Compared with that in NC mimic-transfected cells, the luciferase activity in RUFY3-WT-transfected cells was reduced but was not significantly altered in RUFY3-MUT-transfected cells (Fig. 4O). To further explore the biological regulatory mechanism of this circRNA, we evaluated whether circSORBS1 binds directly to RUFY3 mRNA. Combined with the above bioinformatics analysis results, the results of the RAP assay using circSORBS1-specific desulfated biotin probes suggested the possibility of direct binding between circSORBS1 and RUFY3 mRNA (Fig. 4P, Q). Next, we constructed RUFY3 wild-type and mutant vectors based on the circSORBS1 binding sequence in RUFY3 mRNA and performed dual-luciferase reporter assays (Fig. 4R, S). Overexpression of circSORBS1 decreased luciferase activity in the RUFY3-WT group compared with that in the transfected vector group, whereas overexpression of RUFY3-MUT did not significantly change luciferase activity; thus, these results indicate the direct binding of circSORBS1 to RUFY3 mRNA (Fig. 4T). Subsequently, we treated cell lines with stable circSORBS1 overexpression via Act D, and the results showed that the half-life of RUFY3 mRNA was longer in the circSORBS1 overexpression group than in the control group, suggesting that circSORBS1 enhances RUFY3 mRNA stability (Fig. 4U). Finally, Western blot analysis revealed that RUFY3 protein expression was reduced after transient silencing of circSORBS1 but increased after transient overexpression of circSORBS1, consistent with the above alterations in mRNA levels (Fig. 4V, W). In summary, circSORBS1 has dual regulatory effects, acting as a miR-6779-5p sponge to indirectly regulate RUFY3 mRNA expression and directly binding to RUFY3 mRNA to increase its stability and promote RUFY3 protein expression.

### circSORBS1 regulates apoptosis via the RUFY3/YWHAE pathway

**Fig. 5:**
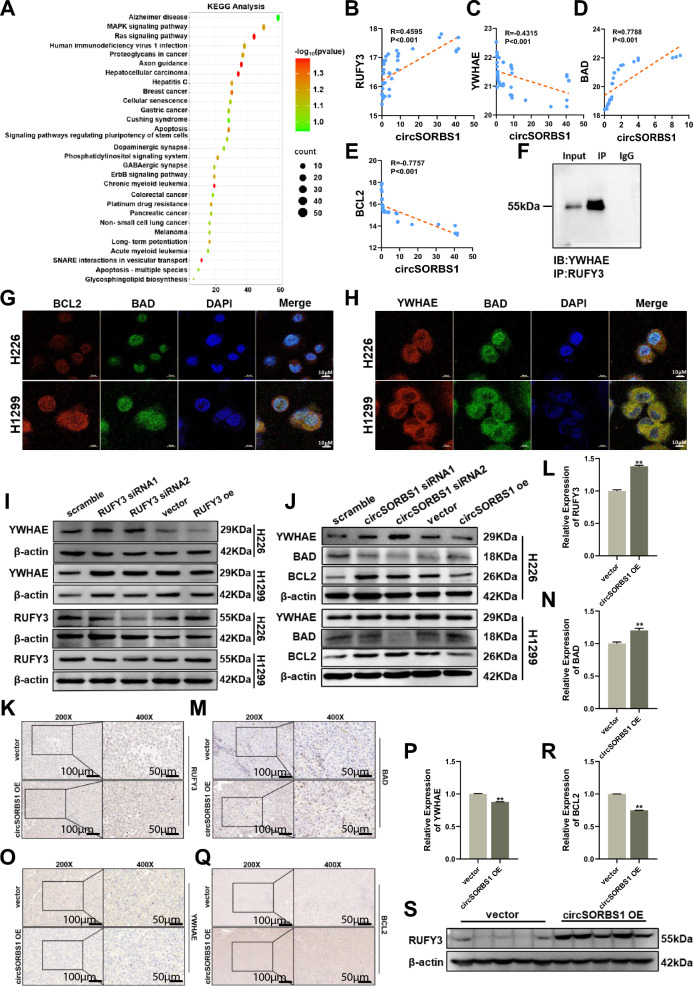
circSORBS1 regulates apoptosis via the RUFY3/YWHAE pathway. **A** KEGG pathway analysis of genes downstream of RUFY3. **B–E** Correlation analysis of circSORBS1 with RUFY3 and key proteins of the apoptotic pathways YWHAE, BAD, and BCL2 in the UCSC database. **F** Results of the Western blot analysis for Co-IP experiments. **G** IF was used to detect the colocalization of the BCL2 protein with the BAD protein. **H** IF was used to detect the colocalization of YWHAE with the BAD protein. **I** Western blot analysis of YWHAE and RUFY3 protein expression after transient RUFY3 silencing and overexpression. **J** Western blot analysis of YWHAE and RUFY3 protein expression after transient circSORBS1 silencing and overexpression. **K–R** Immunohistochemistry was performed to detect the protein expression levels of RUFY3, YWHAE, BAD, and BCL2 in nude mouse tumours. **S** Western blot analysis of RUFY3 protein expression in the tumour tissue of nude mice

Through the above experiments, we found that circSORBS1 regulates RUFY3 protein expression through a dual regulatory mechanism. To further explore the regulatory mechanism of circSORBS1 in lung cancer development, we investigated the signalling pathways downstream of RUFY3 (Fig. 5A). First, after prediction via the online PrePPI database, we found that RUFY3 can bind to key proteins in multiple tumour-related pathways. Moreover, the abovementioned KEGG pathway analysis based on circSORBS1, which linked RUFY3 to the apoptotic signalling pathway, indicated that RUFY3 may bind to YWHAE (Figure S2E). Therefore, we performed Co-IP experiments and found that the YWHAE protein was significantly enriched in the RUFY3 precipitate, suggesting that RUFY3 can bind to YWHAE (Fig. 5F). Western blot analysis revealed that after transient silencing of RUFY3, the expression of YWHAE increased, while after transient overexpression of RUFY3, YWHAE protein expression decreased (Fig. 5I, Fig. S3A, E–F). Finally, by querying the online KEGG database, we found that YWHAE could regulate apoptosis through the classical BAD/BCL2 apoptotic pathway. Subsequently, by performing a raw letter analysis, we found that circSORBS1 was correlated with RUFY3, YWHAE, BCL2 and BAD (Fig. 5B–E). Further Western blot analysis revealed that after transient silencing of circSORBS1, the expression of YWHAE and BCL2 increased, while the expression of BAD decreased; however, after transient overexpression of circSORBS1, the expression of YWHAE and BCL2 decreased, and the expression of BAD increased (Fig. 5J, Fig. S3B–D). Moreover, the results of IF experiments revealed that BAD fluorescence was attenuated and BCL2 fluorescence was enhanced after silencing circSORBS1, while BAD fluorescence was enhanced and BCL2 fluorescence was attenuated after overexpressing circSORSB1, consistent with the results of the above correlation analyses and previously reported studies (Figure S3G and S3H). Next, we performed IF colocalization analysis and verified that YWHAE could activate the BAD/BCL2 signalling pathway (Fig. 5G, H). Finally, using IHC analysis, we found that stable overexpression of circSORBS1 resulted in decreased expression of YWHAE and BCL2 and increased expression of RUFY3 and BAD, consistent with the above in vitro results (Fig. 5K–R). Western blot analysis revealed that the expression level of RUFY3 in the nude mouse tumours was consistent with that in the cells (Fig. 5S). These results indicate that circSORBS1 can promote apoptosis through the RUFY3/YWHAE pathway.

### circSORBS1 inhibits lung cancer development through the RUFY3/YWHAE/BAD/BCL2 pathway

**Fig. 6:**
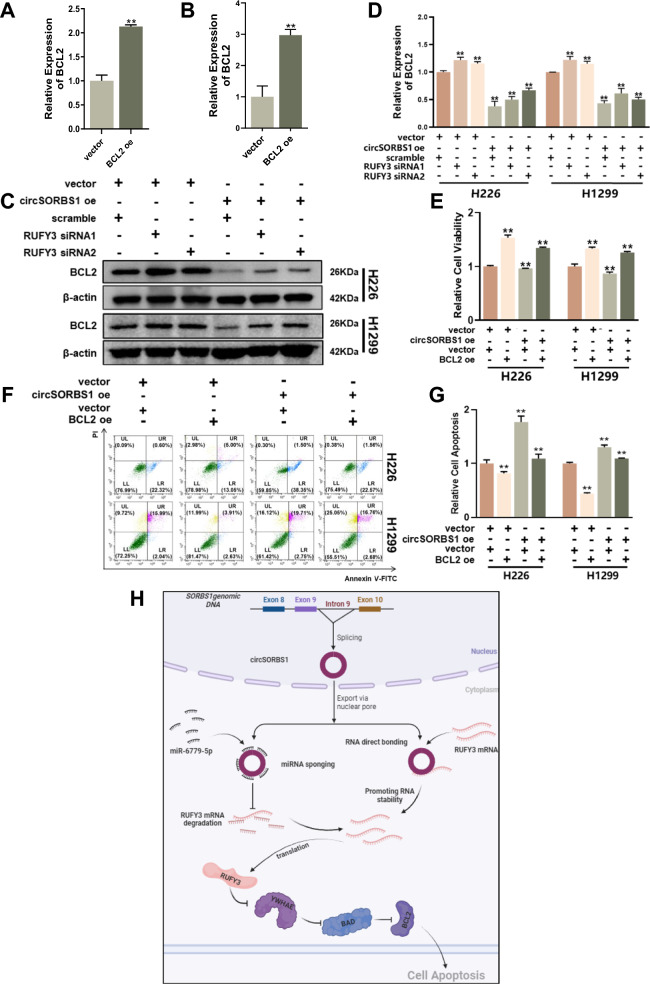
circSORBS1 inhibits lung cancer development through the RUFY3/YWHAE/BAD/BCL2 pathway. **A**, **B** qPCR detection of the transient overexpression efficiency of BCL2 mRNA in H226 versus H1299 cells. **C**, **D**. Western blot analysis of YWHAE protein expression after transient silencing and overexpression of RUFY3 mRNA and grey value analysis. **E** A CCK-8 assay was used to detect the viability of H1299 and H226 cells after transfection with BCL2. **F**, **G** Flow cytometry was used to detect the apoptotic capacity after BCL2 backfilling. **H** circSORBS1 acts as a miR-6779-5p sponge and indirectly inhibits RUFY3 mRNA degradation, directly binds to RUFY3 mRNA and enhances its stability, which in turn increases RUFY3 protein expression, activates the YWHAE/BAD/BCL2 apoptotic signalling pathway, and inhibits lung cancer progression

The above findings suggest that circSORBS1 can affect RUFY3 protein expression through a dual regulatory mechanism, which in turn promotes apoptosis by regulating the BAD/BCL2 pathway through YWHAE. Therefore, we attempted to elucidate the mechanism by which circSORBS1 inhibits lung cancer development by regulating the RUFY3/YHWAE/BAD/BCL2 pathway through rescue experiments. First, we evaluated the BCL2 overexpression efficiency in H226 and H1299 cells (Fig. 6A, B). Subsequently, to more comprehensively understand the mechanism by which circSORBS1 regulates apoptosis, we transiently silenced RUFY3 in H1299 cells stably overexpressing circSORBS1, and Western blot analysis revealed a reversal of the trend towards elevated BCL2 protein expression (Fig. 6C, D). The CCK-8 assay showed that overexpression of circSORBS1 decreased cell viability, overexpression of BCL2 promoted cell viability, and simultaneous overexpression of circSORBS1 and BCL2 reversed the trend towards increased cell viability after overexpression of BCL2 (Fig. 6E). Subsequent evaluation of apoptosis by flow cytometry revealed that overexpression of circSORBS1 promoted apoptosis and that overexpression of BCL2 inhibited apoptosis; moreover, when circSORBS1 was simultaneously overexpressed with BCL2, the trend towards decreased apoptotic activity due to overexpression of BCL2 was reversed (Fig. 6F, G). In summary, we elucidated that circSORBS1 activates the RUFY3/YWHAE/BAD/BCL2 signalling pathway through both a sponging mechanism and direct mRNA binding, ultimately inhibiting the development of lung cancer.

## Discussion

With the development of high-throughput sequencing technology, an increasing number of circRNAs have been identified [12]. circRNAs are widely expressed in human tissues at levels that may be much greater than those of linear parental genes, and compared with those of other ncRNAs, the expression of circRNAs in cells, tissues or body fluids is more stable and specific. Therefore, circRNAs have potential as biomarkers for early tumour detection and evaluation [22, 23]. For example, plasma exosomal circLPAR1 is significantly decreased in colorectal cancer [24]. However, the function of circRNAs in lung cancer has not been fully elucidated. In our study, a differential expression profile of circRNAs in lung cancer was constructed through high-throughput RNA sequencing, and we identified the circRNA circSORBS1, which has not been previously described in lung cancer. Moreover, qPCR revealed that circSORBS1 was significantly downregulated in lung cancer tissues, blood and cell lines. It has been recently shown that circSORBS1 production is regulated by QKI and RBM20 in mice [25, 26]. Qingyao Zhao et al. reported that circSORBS1 adsorbs miR-345-3p in porcine small intestinal epithelial cells, thereby improving cell adhesion [27]. Although circRNAs are given the same name in different species, their sequences and functions differ significantly. These differences mainly arise from evolutionary processes between species, including factors such as mutation, natural selection and genetic drift. The function of circSORBS1 in human lung cancer remains unknown. Therefore, we conducted in vitro and in vivo studies on human samples, and the experimental results showed that circSORBS1 can inhibit various aspects of lung cancer development, including cell viability, proliferation, apoptosis and invasion.

Currently, circRNA studies have focused mainly on their function as a miRNA sponge, while other biological functions and potential molecular mechanisms still need to be further investigated. Our group has previously studied the direct binding of circRNAs to mRNAs [15, 28, 29]. On this basis, we investigated whether circSORBS1 can directly bind to mRNAs to regulate their protein expression while indirectly inhibiting mRNA degradation by acting as a miRNA sponge. To this end, we performed the following additional experiments. circRNAs are widely found in human tissue cells and are mainly enriched in the cytoplasm and, to a lesser extent, in the nucleus [14]. We found that circSORBS1 is mainly localized in the cytoplasm. circRNAs in the nucleus mainly function by influencing the transcriptional regulation of parental genes [30], whereas circRNAs in the cytoplasm mainly function through mechanisms such as miRNA sponging and interactions with proteins [31]. Through bioinformatics analysis (*cat*RAPID), we found that circSORBS1 may bind to the AGO2 protein. Therefore, we constructed a ‘circSORBS1-miRNA‒mRNA’ axis for the experiments. By predicting the potential miRNA targets of circSORBS1, we confirmed that miR-6779-5p is a target of circSORBS1 via RAP and dual luciferase assays. miRNAs are a class of noncoding RNAs with lengths of 22–25 nucleotides that inhibit gene expression by targeting the 3′UTR of mRNAs after transcription. Previous studies have shown that miR-6779-5p is upregulated in glioblastoma and significantly contributes to its development [32], but studies of miR-6779-5p in lung cancer have not been reported. We predicted the downstream target genes of miRNAs and identified RUFY3 as a downstream target of miR-6779-5p by bioinformatics analysis, RAP assays and dual luciferase reporter gene assays. RUFY3 encodes a protein that contains the structural domains of RPIP8, UNC-14, and NESCA and has been reported to promote cell migration and invasion in gastric and hepatocellular carcinomas [33, 34], but this phenomenon has not been reported in lung cancer studies. Using a dual-luciferase reporter gene assay, we predicted the binding site of circSORBS1 for RUFY3 mRNA and confirmed its direct binding. For a more comprehensive understanding of the regulatory mechanism of circRNAs, we will explore the primary and secondary relationships of their dual regulatory mechanisms and their synergistic or competitive roles in future studies.

Abnormal expression of circRNAs is closely related to the occurrence and development of lung cancer [35–37]; however, its mechanism of action has not been fully elucidated. In lung cancer, circRNAs mainly regulate tumour processes by regulating cell viability [38], proliferation [39], apoptosis [40], migration [41] and invasion [37]. In this study, we found that circSORBS1 regulates RUFY3 mRNA expression and that the RUFY3 protein targets the YWHAE protein, which in turn affects the level of apoptosis in tumour cells. Currently, there are few studies on RUFY3, and the function of RUFY3 in lung cancer and its mechanism have not been fully elucidated. In lung cancer, we were the first to find that RUFY3 interacts with YWHAE. Protein interactions can be regulated by various modifications [42], such as phosphorylation [43] and acetylation [44, 45], which affect protein charge and structure; ubiquitination [46], which marks protein degradation; methylation, which alters protein activity and stability; and glycosylation [47], which affects protein stability and function. In future studies, we will further clarify the binding domains between RUFY3 and YWHAE and the types of modifications affecting the relationship between RUFY3 and YWHAE. YWHAEs belong to the 14-3-3 protein family, a group of highly conserved proteins involved in a wide range of important cellular processes, such as protein transport, signalling, apoptosis, and cell cycle regulation [48–50]. Currently, the 14-3-3 protein family is mainly focused on 14-3-3 Sigma (SFN), while the 14-3-3 Epsilon (YWHAE) family has been studied relatively little. YWHAE proteins are structurally very similar to SFN proteins, both consisting of nine conserved α-helical structures forming a cup-shaped trimer, a structure that allows them to bind to a wide range of proteins and influence their activity and function. In contrast to SFN, which is mainly expressed in the nervous system [51], YWHAE is widely distributed in various tissues. Therefore, YWHAE is highly valuable for the study of multiple cancers. Numerous studies have shown that YWHAE plays an important role in the regulation of apoptosis in tumour cells. YWHAE is involved in tumour progression mainly by interacting with the BCL2 protein family [52, 53], affecting the activity of caspase family enzymes, and regulating the p53 signalling pathway [54, 55]. Through bioinformatics analysis, we found that the downregulated gene circSORBS1 in lung cancer is closely related to the BCL2 protein family. Therefore, combined with related reports, we have focused mainly on the classical YWHAE/BAD/BCL2 apoptosis signalling pathway in our subsequent studies. In this study, the novel finding that RUFY3, which is regulated by circSORBS1, targets YWHAE and thus affects the BCL2 apoptosis signalling pathway provides new clues for a deeper understanding of the mechanism regulating apoptosis and is expected to provide new strategies for the treatment of lung cancer.

In summary, in this study, we have proposed a novel model of circRNA regulation in which circSORBS1 acts as a miR-6779-5p sponge to indirectly regulate RUFY3 mRNA, while circSORBS1 directly binds to RUFY3 mRNA via a dual regulatory mechanism. In addition, we found that in lung cancer, low expression of circSORBS1 was significantly correlated with tumour stage, and that circSORBS1 can be detected in plasma, indicating its potential value as a diagnostic target for lung cancer. By exploring the underlying mechanism, we found that circSORBS1 regulates RUFY3 expression directly and indirectly, activates the YWHAE/BAD/BCL2 apoptosis signalling pathway, and ultimately inhibits the development of lung cancer. This study not only enriches our knowledge of circRNA regulatory mechanisms but also provides new insights into the epigenetic mechanisms of action that influence tumour cell fate.

### Supplementary Information

Supplementary Material 1: Fig. 1. High-throughput sequencing data analysis. (A) KEGG cnetplot pathway analysis of circRNAs. (B) circSORBS1 KEGG cnetplot pathway analysis. Supplementary Fig. 2. circSORBS1 downstream regulatory mRNA screen. (A) qPCR detection of SORBS1 expression after silencing and overexpressing circSORBS1. (B) Schematic representation of circSORBS1 binding to AGO2.  (C–D) Construction of the circSORBS1-miRNA‒mRNA regulatory network. (E) Survival analysis of patients with RUFY3-related lung cancer. (F) Correlation analysis between RUFY3 and YWHAE. (G) Survival analysis of patients with YWHAE-related lung cancer. Supplementary Fig. 3. Regulated protein expression downstream of circSORBS. (A) qPCR detection efficiency after transient silencing and overexpression of RUFY3. (B-D) Western blot analysis of YWHAE, BAD, and BCL2 protein expression and grey value analysis after transient silencing and overexpression of circSORBS1. (E-F) Western blot analysis of YWHAE and RUFY3 protein expression and grey value analysis after transient silencing and overexpression of RUFY3. (G-H) IF was used to detect BAD and BCL2 expression after silencing and overexpressing circSORBS1.

Supplementary Material 2.

Supplementary Material 3.

## Data Availability

Sequencing raw data in this study can be found online from Sequence Read Archive (SRA) (PRJNA1086213).
