# Supplementary figures and images for: circSORBS1 inhibits lung cancer progression by sponging miR-6779-5p and directly binding RUFY3 mRNA
Source: J Transl Med. 2024 Jun 24;22:590. doi: 10.1186/s12967-024-05423-0 (PMC11197270; doi:10.1186/s12967-024-05423-0)

**A**

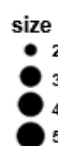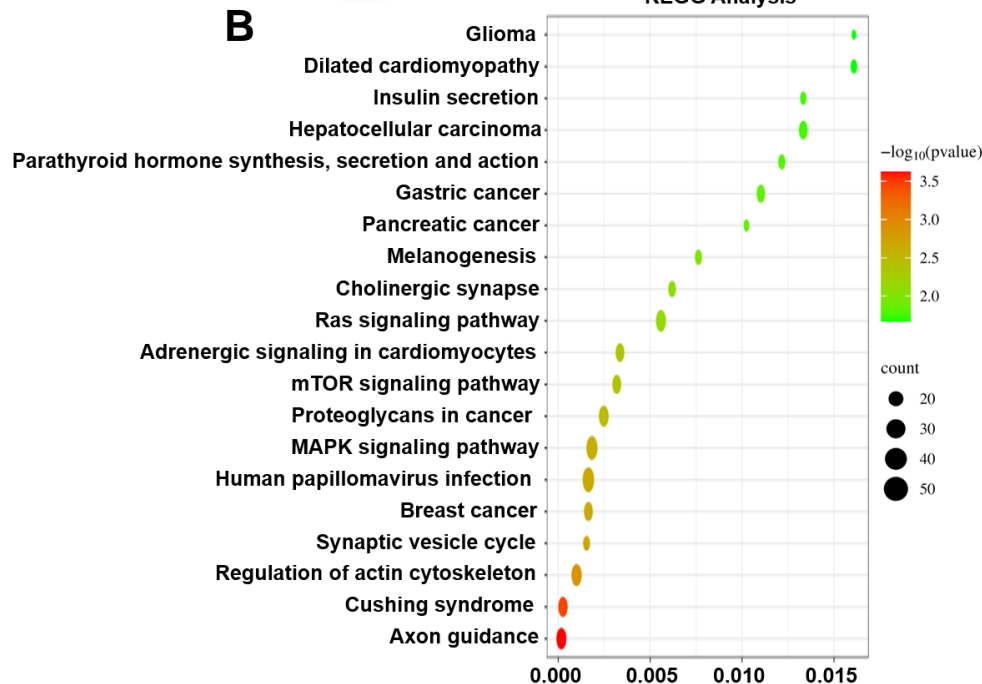

# Supplementary Figure 2

**A**

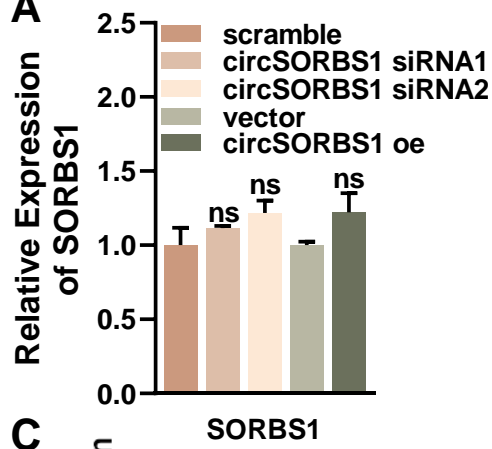

**B**

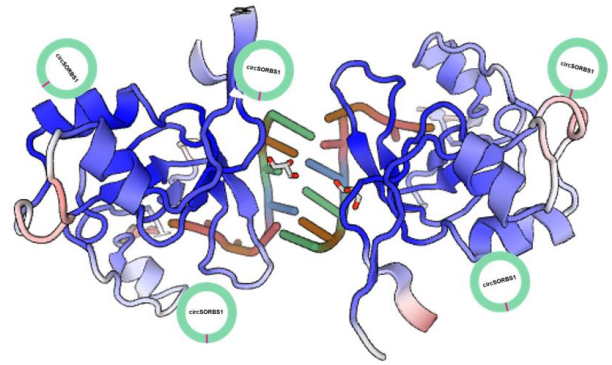

**C**

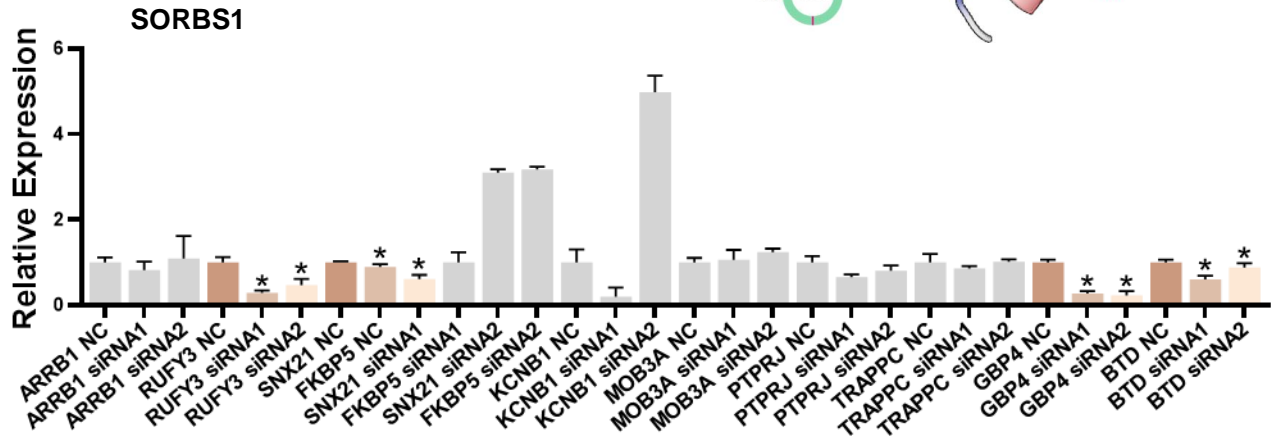

**E**

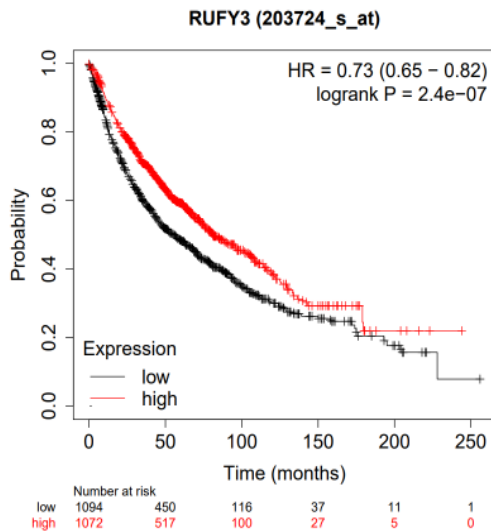

**D**

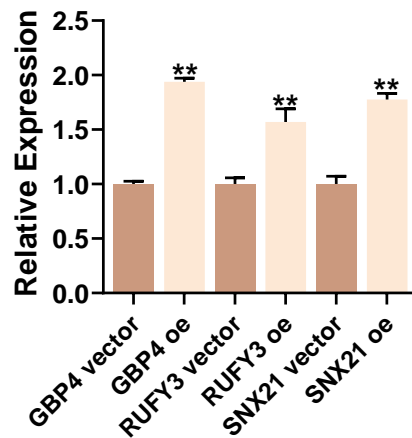

**F**

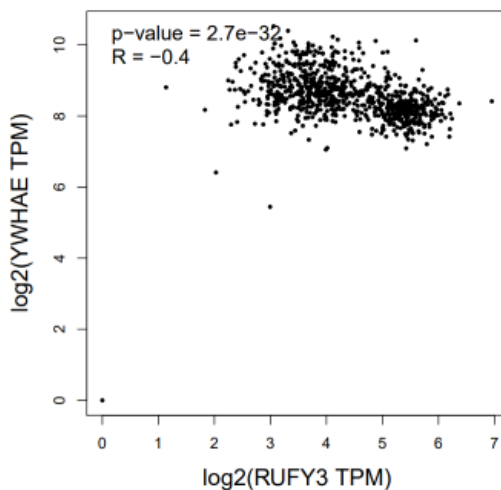

**G**

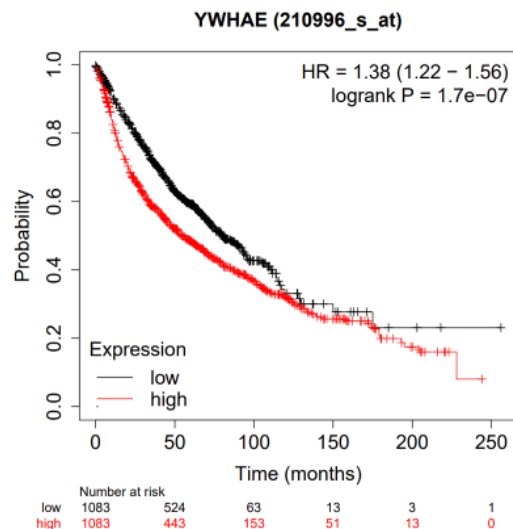

# Supplementary Figure 3

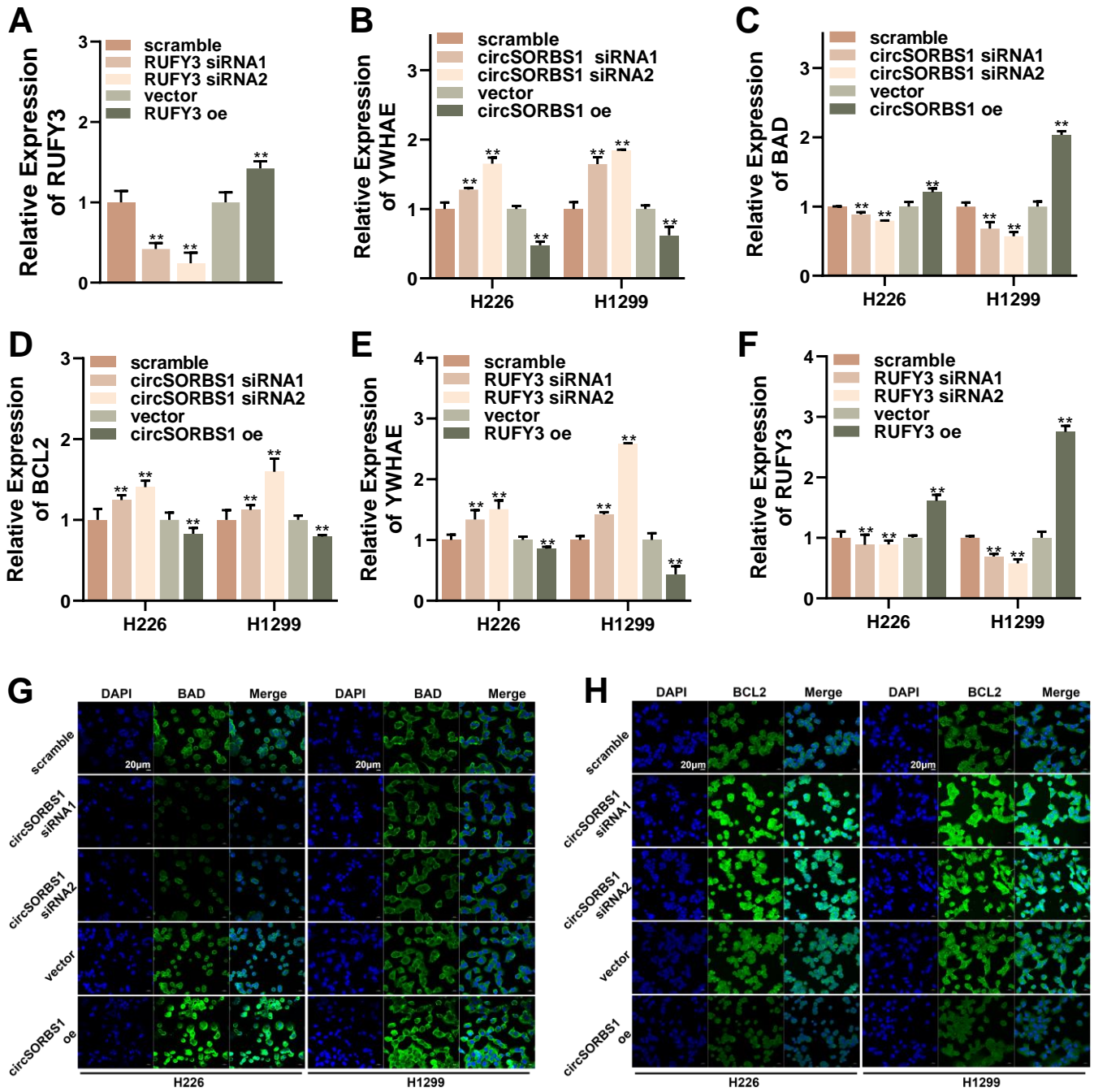

Supplement: Supplementary file 1 — Supplementary Material 1: Fig. 1. High-throughput sequencing data analysis. (A) KEGG cnetplot pathway analysis of circRNAs. (B) circSORBS1 KEGG cnetplot pathway analysis. Supplementary Fig. 2. circSORBS1 downstream regulatory mRNA screen. (A) qPCR detection of SORBS1 expression after silencing and overexpressing circSORBS1. (B) Schematic representation of circSORBS1 binding to AGO2. (C–D) Construction of the circSORBS1-miRNA‒mRNA regulatory network. (E) Survival analysis of patients with RUFY3-related lung cancer. (F) Correlation analysis between RUFY3 and YWHAE. (G) Survival analysis of patients with YWHAE-related lung cancer. Supplementary Fig. 3. Regulated protein expression downstream of circSORBS. (A) qPCR detection efficiency after transient silencing and overexpression of RUFY3. (B-D) Western blot analysis of YWHAE, BAD, and BCL2 protein expression and grey value analysis after transient silencing and overexpression of circSORBS1. (E-F) Western blot analysis of YWHAE and RUFY3 protein expression and grey value analysis after transient silencing and overexpression of RUFY3. (G-H) IF was used to detect BAD and BCL2 expression after silencing and overexpressing circSORBS1. [file 12967_2024_5423_MOESM1_ESM.pdf]
