# Supplementary material for: circSORBS1 inhibits lung cancer progression by sponging miR-6779-5p and directly binding RUFY3 mRNA
Source: J Transl Med. 2024 Jun 24;22:590. doi: 10.1186/s12967-024-05423-0 (PMC11197270; doi:10.1186/s12967-024-05423-0)
Supplement: Supplementary file 2 — Supplementary Material 2. [file 12967_2024_5423_MOESM2_ESM.docx]

**Supplementary Information**

**Table S1. Analysis of clinical case information on lung cancer in circSORBA1**

| **Variables** | **Cases**  **(total n = 79)** | | **circSORBS1**  **High (n) Low (n)** | | **χ^2^** | **P-value** |
| --- | --- | --- | --- | --- | --- | --- |
| **All cases** | 79 | | 30 | 49 | — | — |
| **Age** |  |  | | | | |
| ≥60 | 34 | | 14 | 20 | 0.260 | 0.610 |
| <60 | 45 | | 16 | 29 |  |  |
| **Gender** |  |  | | | | |
| Male | 46 | | 17 | 29 | 0.048 | 0.826 |
| Female | 33 | | 13 | 20 |  |  |
| **T stage** |  |  | | | | |
| T1-2 stage | 65 | | 21 | 44 | 5.001 | 0.025 |
| T3-4 stage | 14 | | 9 | 5 |  |  |
| **Lymph node metastasis** |  |  | | | | |
| positive | 11 | | 4 | 7 | 2.512 | 0.113 |
| negative | 68 | | 26 | 42 |  |  |
| **TNM stage** |  |  | | | | |
| I stage | 62 | | 22 | 40 | 0.759 | 0.384 |
| II and III stage | 17 | | 8 | 9 |  |  |
| **Smoking** |  |  | | | | |
| Yes | 15 | | 3 | 12 | 2.540 | 0.111 |
| No | 64 | | 27 | 37 |  |  |
