# Supplementary material for: circSORBS1 inhibits lung cancer progression by sponging miR-6779-5p and directly binding RUFY3 mRNA
Source: J Transl Med. 2024 Jun 24;22:590. doi: 10.1186/s12967-024-05423-0 (PMC11197270; doi:10.1186/s12967-024-05423-0)
Supplement: Supplementary file 3 — Supplementary Material 3. [file 12967_2024_5423_MOESM3_ESM.doc]

**Supplementary Information**

**Table S2. Information of the qPCR primer sequences and silencing RNA sequences**

| **qPCR primer name** | **Sequence (5’-3’)** |
| --- | --- |
| circSORBS1 (Forward) | AAGAAATGACTGGGAACCCCC |
| circSORBS1 (Reverse) | AGGACTGGAATCCATCTTTTCGT |
| SORBS1 (Forward) | ATTCCCAAGCCTTTCCATCAG |
| SORBS1 (Reverse) | TTTTGCTGTTCTCGATTGTGTTG |
| circSORBS1 OE (Forward) | CGCGGATCCGATTCCAGTCCTCTACTAAATGAAGT |
| circSORBS1 OE (Reverse) | CCGGAATTCCATCTTTTCGTTGGTCAGAACGGAAG |
| RUFY3 (Forward) | GGAAGACGGCAGCCTAACAA |
| RUFY3 (Reverse) | CAGGGATTGCAAACTCGCTC |
| GBP4 (Forward) | GGCTGCTAAAACACAAGCTGAA |
| GBP4 (Reverse) | AAGCCCCAGGTAGAGTGACA |
| SNX21 (Forward) | AGAAGTCCCGGAACACCTTG |
| SNX21 (Reverse) | GGCGAGGGTGTAGAGTTGGA |
| ARRB1 (Forward) | AAAGGGACCCGAGTGTTCAAG |
| ARRB1 (Reverse) | CGTCACATAGACTCTCCGCT |
| PRR12 (Forward) | GCGGGATGGAGTTACGAGAG |
| PRR12 (Reverse) | GGCGGTGTAAGATGTCCGTC |
| NSD (Forward) | AGAGGACGCTGAGAAGGATG |
| NSD (Reverse) | CCGTAGATGATGGCGATAAACTC |
| H6PD (Forward) | GCAGAGCACAAGGATCAGTTC |
| H6PD (Reverse) | GGCAGCTACTGTTGATGTTGC |
| FKBP5 (Forward) | AATGGTGAGGAAACGCCGATG |
| FKBP5 (Reverse) | TCGAGGGAATTTTAGGGAGACT |
| KCNB1 (Forward) | ACTCTGGCGTACCCTGGAC |
| KCNB1 (Reverse) | GTCGTCGAGGCTGTAGTCATC |
| MOB3A (Forward) | CCCCAAGCGCAAGTTTGAG |
| MOB3A (Reverse) | TTGACGCGGTTAAAGAAGTCC |
| PTPRJ (Forward) | GGCACCCCTAGTCCAATTCC |
| PTPRJ (Reverse) | TCCCATTAGATCCTTGTTCAGGT |
| TRAPPC (Forward) | TCACCCTGACCTATGGTGC |
| TRAPPC (Reverse) | GCCGGACTCCAATGTTAAAGC |
| BTD (Forward) | GGCATGGCTACCTGTGAGATG |
| BTD (Reverse) | GGCCAAAATGCTTGCCTTCT |
| ELN (Forward) | GCAGGAGTTAAGCCCAAGG |
| ELN (Reverse) | TGTAGGGCAGTCCATAGCCA |
| BCL2 (Forward) | TTGCCAGCCGGAACCTATG |
| BCL2 (Reverse) | CGAAGGCGACCAGCAATGATA |
| GAPDH (Forward) | ATCAATGGAAATCCCATCACCA |
| GAPDH (Reverse) | GACTCCACGACGTACTCAGCG |
| U6 (Forward) | GGAACGATACAGAGAAGATTAGC |
| U6 (Reverse) | TGGAACGCTTCACGAATTTGCG |
| **siRNA name** | **Sequence (5’-3’)** |
| circSORBS1 siRNA1 (sense) | CCAACGAAAAGAUGGAUUC dTdT |
| circSORBS1 siRNA2 (sense)  scramble (sense)  scramble (antisense) | CAAUCGAGAACUUCCUCAC dTdT  UUCUCCGAACGUGUCACGUTT  ACGUGACACGUUCGGAGAATT |
| **miRNA name** | **Sequence (5’-3’)** |
| hsa-miR-6779-5p | TGGGAGGGGCTGGGTTTG |
| hsa-miR-4507 | CUGGGUUGGGCUGGGCUGGG |
| **FISH probe** | **Sequence (5’-3’)** |
| circSORBS1-FISH | TTCTGACCAACGAAAAGATGGATTCCAGTCCTCTACTAAA |
| circSORBS1-RAP | AAGTCTTCCGTTCTGACCAACGAAAAGATGGATTCCAGTCCTCTACTAAATGAA |
|  |  |
|  |  |
|  |  |
|  |  |
|  |  |
